# Reliability, Validity, and Diagnostic Accuracy of the Persian Six‐Item Cognitive Impairment Test (6‐CIT) in Older Adult Outpatients

**DOI:** 10.1002/brb3.71818

**Published:** 2026-09-30

**Authors:** Seyed Mohammad Mahdi Sadeghi, Amirreza Esmaeili, Maryam Pourshams, Vahid Saeedi, Seyed Kazem Malakouti, Leila Kamalzadeh

**Affiliations:** ^1^ Geriatric Mental Health Research Center, Department of Psychiatry, School of Medicine Iran University of Medical Sciences Tehran Iran; ^2^ Department of Clinical Health Psychology, School of Behavioral Science and Mental Health Iran University of Medical Science Tehran Iran; ^3^ Department of Psychiatry Golestan Hospital, Ahvaz Jundishapur University of Medical Sciences Ahvaz Iran; ^4^ Pediatric Growth and Development Research Center, School of Medicine Iran University of Medical Sciences Tehran Iran

**Keywords:** 6‐CIT, cognitive screening, dementia, mild cognitive impairment

## Abstract

**Introduction:**

Early detection of neurocognitive disorders is crucial for timely intervention in older adults. The Six‐Item Cognitive Impairment Test (6‐CIT) is a brief screening tool designed for rapid cognitive assessment, but a validated Persian version has not been available to date. This study aimed to translate, culturally adapt, and psychometrically evaluate the Persian version of the 6‐CIT among Iranian older adults.

**Methods:**

In this prospective cross‐sectional psychometric validation and diagnostic accuracy study, 180 participants aged ≥ 60 years were recruited from outpatient geriatric clinics and categorized into three equal groups: normal cognition, Mild Cognitive Impairment (MCI), and dementia. Diagnoses were established by a geriatric psychiatrist using DSM‐5 criteria, and severity was staged using the Clinical Dementia Rating (CDR) criteria. Reliability was assessed through internal consistency (Cronbach's *α*) and test–retest stability (intraclass correlation coefficient, ICC 3,1). Validity was examined via convergent validity with the Mini‐Mental State Examination (MMSE) and exploratory principal component analysis (PCA). Diagnostic accuracy, sensitivity, and specificity were evaluated using receiver operating characteristic (ROC) analysis and Youden's J index.

**Results:**

The Persian 6‐CIT demonstrated good internal consistency (Cronbach's *α* = 0.836) and strong test–retest reliability (ICC = 0.849). A strong negative correlation with the MMSE supported convergent validity (*ρ* = –0.919, *p* < 0.001). PCA supported a unidimensional structure explaining 71.72% of the total variance. ROC analysis for dementia yielded an AUC of 0.984 (95% CI: 0.961–1.000); a cutoff score of ≥ 11 provided 96.7% sensitivity and 85.8% specificity, while a cutoff of ≥ 14 maximized overall mathematical accuracy (93.3% sensitivity, 94.2% specificity). Furthermore, diagnostic accuracy for MCI was also high (AUC = 0.976, 95% CI: 0.947–0.997), with an optimal cutoff of ≥ 2 yielding 96.7% sensitivity and 96.7% specificity.

**Conclusions:**

The Persian 6‐CIT is a brief, reliable, and valid instrument for screening both MCI and dementia among Iranian older adults in specialized outpatient settings.

## Graphical abstract

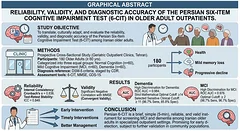

## 1 Introduction

Aging is a complex process often accompanied by a higher risk of neurocognitive disorders such as Mild Cognitive Impairment (MCI) and dementia (Livingston et al. 2024; Kamalzadeh et al. 2019). Globally, over 78 million people are projected to be living with dementia by 2030 (Alzheimer's Disease International [ADI] 2024). Similarly, MCI affects approximately 15.5% of people over the age of 60 or higher depending on population estimates (Song et al. 2023). Early and accurate detection of cognitive decline is therefore critical, as timely interventions may delay or prevent further decline and improve patient outcomes. However, diagnosing cognitive impairment in its early stages is challenging due to symptom heterogeneity and the influence of factors such as age, education, linguistic background, and culture on cognitive testing (Brickman et al. 2006). The Mini‐Mental State Examination (MMSE) relies heavily on formal education and verbal skills, while the Montreal Cognitive Assessment (MoCA), despite adding executive tasks, still exhibits strong cultural and educational biases if not rigorously normed. Conversely, the 6‐CIT, by relying on fundamental orientation and robust working memory tasks, potentially mitigates some of these biases, making it highly suitable for diverse clinical populations. These challenges underscore the importance of reliable and valid screening tools to identify neurocognitive disorders in older adults at the earliest possible stage.

Short cognitive screening tests are often the first step in assessing patients with suspected cognitive impairment and can be administered by specialists or primary care physicians (Fowler et al. 2025; Kamalzadeh et al. 2024; Janssen et al. 2017). While common tools like the MMSE and MoCA have well‐established utility, they also possess limitations, such as administration time in ultra‐brief emergency settings, the need for rigorous linguistic adaptation, or—in the case of the MMSE—limited sensitivity to mild impairment (Chun et al. 2021).

The Six‐Item Cognitive Impairment Test (6‐CIT) is a brief screening tool designed for rapid assessment of cognitive status in older adults. It consists of six questions that assess three cognitive domains: orientation, attention/calculation, and short‐term memory (Katzman et al. 1983; Gale and Larner 2017). Administered in under 5 min with minimal training, it has demonstrated a pooled sensitivity of 0.82 and specificity of 0.87 for detecting dementia (Park 2023). Another advantage of the 6‐CIT is that it has demonstrated cross‐cultural adaptability (Apóstolo et al. 2018). These characteristics make the 6‐CIT an attractive option for cognitive screening, especially in time‐pressed primary care or emergency department contexts for detecting chronic cognitive impairment (Salis et al. 2023; O'Caoimh and Molloy 2023; O'Sullivan et al. 2016).

Despite its widespread adaptation into numerous languages, a validated Persian version of the 6‐CIT has not been available for Iranian older adults. Thus, this study aimed to translate the 6‐CIT into Persian and evaluate its psychometric properties and diagnostic accuracy for both MCI and dementia within an outpatient clinical population.

## 2 Materials and Methods

### 2.1 Study Design and Setting

This prospective cross‐sectional psychometric validation and diagnostic accuracy study was conducted from January 2024 to January 2025 in specialized outpatient geriatric clinics (Rasool Akram Hospital and the Memory Clinic of Iran University of Medical Sciences) in Tehran, Iran.

### 2.2 Participants and Clinical Assessment

A total of 220 older adults (≥ 60 years) were initially assessed for eligibility. During the screening phase, 40 individuals were excluded for the following reasons: GDS score ≥ 8 (*n* = 12), lack of adequate collateral information from caregivers (*n* = 15), history of major psychiatric illness (*n* = 8), and incomplete assessment data (*n* = 5). This participant flow yielded a final convenience sample of 180 patients. Participants were explicitly assigned to diagnostic groups by a single, blinded geriatric psychiatrist based on a semi‐structured psychiatric evaluation, collateral history from a reliable informant, and review of medical records applying DSM‐5 criteria for neurocognitive disorders (with MCI corresponding to the DSM‐5 category of Mild Neurocognitive Disorder). The Clinical Dementia Rating (CDR) scale was not used for primary diagnosis but was administered sequentially as an independent clinical staging tool to establish severity: Cognitively Normal Controls (CDR = 0), MCI (CDR = 0.5), and Major Neurocognitive Disorder (dementia, CDR ≥ 1). The diagnostic assessor was strictly blinded to the 6‐CIT results.

Exclusion criteria included major psychiatric illnesses, seizures, severe head trauma, current substance use disorders, or intellectual disability. To prevent depression from confounding cognitive scores (pseudodementia), individuals scoring ≥8 on the 15‐item Geriatric Depression Scale (GDS‐15) were also excluded (19). While depressive symptoms are common in this demographic, this design decision was intended to reduce the potential influence of depressive symptom to ensure cognitive deficits were primarily neurocognitive in origin, though this restricts the clinical spectrum. All participants were required to be fluent in Persian and provide written informed consent (World Medical Association 2013).

### 2.3 Instruments

Demographic and Clinical Questionnaire: A structured questionnaire was administered to collect demographic information (age, sex, marital status, years of education) and relevant medical history. Medical and psychiatric conditions were coded based on the single primary diagnosis to ensure mutually exclusive categories.

6‐CIT: The 6‐CIT is a brief cognitive screening tool comprising six questions that assess orientation, attention/calculation, and short‐term memory. The test yields a weighted score out of 28 points, with higher scores indicating worse cognitive performance (Katzman et al. 1983).

MMSE: The MMSE is a widely used 30‐point cognitive screening tool assessing orientation, registration, attention, recall, and language. The validated Persian version was administered in this study (Folstein et al. 1975; Seyedian et al. 2008).

Geriatric Depression Scale (GDS‐15): The 15‐item GDS is designed to screen for depressive symptoms in older adults. The Persian GDS‐15, with 90% sensitivity and 84% specificity, was used in this study (Malakouti et al. 2006).

### 2.4 Translation and Cultural Adaptation of the 6‐CIT

Following permission from the original developers, a standardized forward–backward translation protocol was implemented. Two independent bilingual clinical psychologists and medical professionals translated the original English 6‐CIT into Persian. Following a reconciliation meeting to resolve discrepancies, two different blinded experts performed backward translation. A multidisciplinary expert panel comprising three geriatric psychiatrists and two clinical neuropsychologists reviewed the final version to calculate the content validity ratio (CVR, based on item necessity using the Lawshe method: 0.60–1.00) and Content Validity Index (scale‐level CVI, based on clarity and relevance, with a score > 0.79 considered excellent: 0.97). Pilot testing via cognitive interviewing was then conducted on 15 Iranian older adults to ensure comprehensibility.

### 2.5 Sample Size

Sample size was determined based on the primary goal of receiver operating characteristic (ROC) curve analysis. To detect an expected AUC of 0.80, compared to a null hypothesis of 0.50 with an alpha of 0.05 and 80% power, approximately 40 participants per group were required. We recruited 60 per group to ensure robust psychometric parameter estimation.

### 2.6 Statistical Analysis

Analyses were performed using Python (Pandas/SciPy) and SPSS version 26.0 (significance at *p* < 0.05). Missing data were handled using listwise deletion (no missing data were present in the final dataset). Data distributions were assessed using the Shapiro–Wilk test. Due to the violation of normality (*p* < 0.05), non‐parametric tests were utilized. Group baseline differences were tested using ANOVA and Chi‐Square tests; ordinal variables (e.g., education) were analyzed using the Kruskal–Wallis test, followed by post hoc pairwise comparisons using Dunn's test with Bonferroni correction for multiple comparisons.

Reliability was assessed via Intraclass Correlation Coefficient (ICC model 3,1: two‐way mixed, single measures, absolute agreement) for test–retest stability (two‐week interval, *n* = 46; Normal = 2, MCI = 24, dementia = 20, recruited as a convenience subsample from consecutive patients attending routine follow‐up visits) and Cronbach's *α* for internal consistency. Construct validity was established via one‐way ANOVA. Internal structural validity was evaluated using principal component analysis (PCA) as an exploratory data reduction technique; no rotation method was applied because only one component with an eigenvalue > 1 was extracted. Convergent validity was quantified using Spearman's correlation (*ρ*) against the MMSE, which was selected because the 6‐CIT scoring system utilizes a weighted ordinal scale. ROC analysis and Youden's J index determined the optimal clinical cutoffs, area under the curve (AUC), sensitivity, and specificity. Additionally, multivariable logistic regression was conducted to assess whether the 6‐CIT diagnostic discrimination remained significant after adjusting for educational level as a potential confounder.

## 3 Results

### 3.1 Sample Characteristics

The 180 participants had a mean age of 71.86 ± 11.23 years. As detailed in Table 1, the three diagnostic groups did not differ significantly regarding age, sex, marital status, past medical history, psychiatric history, and substance use (all *p* > 0.05). Education levels differed significantly (Kruskal–Wallis H = 13.209, *p* = 0.001); post hoc analysis revealed the MCI group had higher education levels, compared to the dementia group, though no significant difference existed between the normal cognition and dementia groups. Significant floor effects were observed in the normal cognition group, where the median 6‐CIT score was 0.

**TABLE 1:** Demographic and clinical characteristics of the study sample by diagnostic group.

Characteristic	Total sample (*N* = 180)	Normal cognition (*n* = 60)	MCI (*n* = 60)	Dementia (*n* = 60)	Statistical test and *p*‐value
**Age (years), mean ± SD**	71.86 ± 11.23	72.00 ± 8.54	69.62±15.32	73.95 ± 8.28	*F* = 2.253, *p* = 0.108
**Sex, *n* (%)**					χ^2^ = 2.340, df = 2, *p* = 0.310
‐ Male	100 (55.6%)	30 (50.0%)	32 (53.3%)	38 (63.3%)	
‐ Female	80 (44.4%)	30 (50.0%)	28 (46.7%)	22 (36.7%)	
**Marital status, *n* (%)**					χ^2^ = 7.234, df = 4, *p* = 0.124
‐ Married	86 (47.8%)	30 (50.0%)	33 (55.0%)	23 (38.3%)	
‐ Single/separated/divorced	27 (15.0%)	9 (15.0%)	11 (18.3%)	7 (11.7%)	
‐ Widowed	67 (37.2%)	21 (35.0%)	16 (26.7%)	30 (50.0%)	
**Education level, *n* (%)**					H(2) = 13.209, *p* = 0.001*
‐ Illiterate	32 (17.8%)	10 (16.7%)	7 (11.7%)	15 (25.0%)	
‐ Under Diploma	76 (42.2%)	26 (43.3%)	20 (33.3%)	30 (50.0%)	
‐ Diploma	35 (19.4%)	12 (20.0%)	13 (21.7%)	10 (16.7%)	
‐ University degree	37 (20.6%)	12 (20.0%)	20 (33.3%)	5 (8.3%)	
**Past medical history, *n* (%)**					χ^2^ = 6.510, df = 10, *p* = 0.771
‐ None	29 (16.1%)	8 (13.3%)	12 (20.0%)	9 (15.0%)	
‐ Metabolic (DM, obesity, etc.)	68 (37.8%)	24 (40.0%)	19 (31.7%)	25 (41.7%)	
‐ Cardiovascular (HTN, IHD)	48 (26.7%)	13 (21.7%)	17 (28.3%)	18 (30.0%)	
‐ Cerebrovascular	14 (7.8%)	5 (8.3%)	6 (10.0%)	3 (5.0%)	
‐ Osteoarthritis	13 (7.2%)	6 (10.0%)	3 (5.0%)	4 (6.7%)	
‐ Other medical comorbidities	8 (4.4%)	4 (6.7%)	3 (5.0%)	1 (1.7%)	
**Past psychiatric history, *n* (%)**					χ^2^ = 5.007, df = 6, *p* = 0.543
‐ None	85 (47.2%)	28 (46.7%)	31 (51.7%)	26 (43.3%)	
‐ Anxiety disorders	31 (17.2%)	11 (18.3%)	11 (18.3%)	9 (15.0%)	
‐ Depressive disorders	25 (13.9%)	4 (6.7%)	7 (11.7%)	4 (6.7%)	
‐ Other psychiatric disorders	39 (21.7%)	17 (28.3%)	11 (18.3%)	21 (35.0%)	
**Substance use history, *n* (%)**					χ^2^ = 3.816, df = 4, *p* = 0.431
‐ None	110 (61.1%)	40 (66.7%)	38 (63.3%)	32 (53.3%)	
‐ Nicotine/tobacco	44 (24.4%)	11 (18.3%)	16 (26.7%)	17 (28.3%)	
‐ Opioid	26 (14.4%)	9 (15.0%)	6 (10.0%)	11 (18.3%)	

*Note*: * Indicates statistical significance at the *α* = 0.05 level. For ordinal comparisons of Education, the Kruskal–Wallis test yielded H(2) = 13.209, *p* = 0.001. Medical and psychiatric history categories reflect the single primary diagnosis.

### 3.2 Psychometric Properties of the Persian 6‐CIT

#### 3.2.1 Reliability

The internal consistency of the Persian 6‐CIT was demonstrated with an overall Cronbach's *α* of 0.836. Item‐total statistics indicated that while most items contributed to the scale's reliability, the deletion of Item 3 (About what Time is it?) would slightly increase the alpha to 0.840 (Table 2). Test–retest reliability was calculated using a single measures, absolute agreement ICC model, yielding a coefficient of 0.849 (95% CI: 0.738–0.915, *p* < 0.001). Furthermore, the expert panel confirmed the content validity of the Persian version, yielding a CVR ranging from 0.60 to 1.00 across items, and an excellent scale‐level CVI of 0.97.

**TABLE 2:** Item‐total statistics and internal consistency of the 6‐CIT.

Item	Scale mean if item deleted	Scale variance if item deleted	Corrected item‐total correlation	Cronbach's alpha if item deleted
Q1 ‐ What Year is it?	8.53	59.055	0.741	0.787
Q2 ‐ What Month is it?	8.86	67.215	0.623	0.816
Q3 ‐ About What Time is it?	9.12	72.622	0.456	0.84
Q4 ‐ Count Backward	8.6	62.487	0.739	0.794
Q5 ‐ Months in Reverse	8.06	59.901	0.702	0.794
Q6 ‐ Repeat Address	5.71	38.654	0.746	0.831

#### 3.2.2 Validity

Construct validity (known‐groups and structural): A stepwise progression of impairment was observed across groups: normal cognition (mean = 0.20 ± 1.09), MCI (mean = 8.73 ± 3.88), and dementia (mean = 20.33 ± 5.33). One‐way ANOVA revealed statistically significant differences among the groups (*F*(2, 177) = 411.95, *p* < 0.001), which were supported by Tukey's HSD post hoc tests. To assess internal structure, PCA was performed. The Kaiser–Meyer–Olkin measure verified the sampling adequacy (KMO = 0.830), and Bartlett's test of sphericity was significant (χ^2^ = 524.02, df = 15, *p* < 0.001). The analysis extracted a single component with an eigenvalue > 1, explaining 71.72% of the total variance.

Convergent validity: A significant negative correlation was found between the 6‐CIT and MMSE scores (*ρ* = –0.919, *p* < 0.001), providing evidence of convergent validity.

### 3.3 Diagnostic Accuracy for Dementia and MCI Screening

ROC curve analysis evaluating the 6‐CIT's ability to differentiate dementia from non‐dementia cases yielded an AUC of 0.984 (95% CI: 0.961–1.000, *p* < 0.001). Based on Youden's J index, the strict mathematical optimal cut‐off was ≥ 14 (Youden's J = 0.875; Sensitivity = 93.3%, Specificity = 94.2%). However, to maximize case‐finding utility in a screening context, a more sensitive proposed clinical cut‐off of ≥ 11 was selected, providing a higher sensitivity of 96.7% (95% CI: 88.6%–99.1%) and a specificity of 85.8% (95% CI: 78.5%–91.0%). At this cutoff, the classification frequencies were 58 true positives, 103 true negatives, 17 false positives, and 2 false negatives.

Furthermore, ROC analysis for identifying MCI versus normal cognition demonstrated high discrimination with an AUC of 0.976 (95% CI: 0.947–0.997, *p* < 0.001). The optimal cut‐off score for detecting MCI was ≥ 2, yielding a sensitivity of 96.7% (95% CI: 88.6%–99.1%) and specificity of 96.7% (95% CI: 88.6%–99.1%). The classification frequencies for MCI were 58 true positives, 58 true negatives, two false positives, and two false negatives.

To assess the impact of education, a stratified ROC analysis was conducted. The ≥ 11 cut‐off maintained robust diagnostic performance across both educational strata: Low Education (< Diploma) demonstrated 96.0% sensitivity and 87.5% specificity, while High Education (≥ Diploma) demonstrated 100.0% sensitivity and 83.3% specificity. To ensure that the diagnostic discrimination was not solely driven by educational level differences between groups, a multivariable logistic regression analysis was conducted. The model demonstrated that the 6‐CIT total score remained a significant independent predictor of diagnostic status (Odds Ratio = 1.95, 95% CI: 1.55–2.45, *p* < 0.001) even after adjusting for the educational level.

## 4 Discussion

In this study, we translated the 6‐CIT into Persian and evaluated its psychometric properties and diagnostic accuracy in a clinical sample of older Iranian adults. The findings demonstrate that the Persian 6‐CIT is a reliable and valid instrument for evaluating cognitive impairment, offering adequate discriminatory power for both dementia and MCI.

The internal consistency of the Persian 6‐CIT was acceptable (Cronbach's *α* = 0.836). This value aligns with or exceeds those reported in previous validation studies across different linguistic and cultural contexts, such as Pakistani (Sabzwari et al. 2016) and German (Hessler et al. 2014) samples. However, this variability in *α* should be contextualized; the higher coefficient in our study likely reflects sample heterogeneity, as our clinic‐based sample included definite cases across the cognitive spectrum, whereas routine screening samples often have a restricted range of scores rather than inherently superior psychometric performance. Test–retest reliability provided evidence of short‐term stability over a 2‐week interval (ICC = 0.849).

The very strong inverse correlation with the MMSE (Spearman *ρ* = –0.919) provides evidence of convergence between the two global cognitive measure. Our PCA identified a single‐component structure (explaining 71.72% of variance), which suggests that the Persian items coherently assess general cognitive impairment.

The 6‐CIT scores differed markedly across diagnostic groups, with a stepwise gradient from normal cognition to MCI and dementia. This supports the instrument's ability to distinguish clinically defined groups, although it should be noted that education levels also differed across these groups.

The diagnostic accuracy for differentiating dementia from non‐dementia cases yielded a high AUC of 0.984. While Salis et al. reported an AUC of 0.947 with a cutoff of 8/9 (Salis et al. 2023), and Park's meta‐analysis found a pooled sensitivity of 0.82 and specificity of 0.87 (Park 2023), our results at the clinical screening cutoff of ≥ 11 demonstrated a sensitivity of 96.7% and specificity of 85.8% (with the mathematical Youden cutoff being ≥ 14). While a cut‐off of ≥ 14 maximized overall mathematical accuracy based on Youden's index, screening instruments in geriatric care must prioritize high sensitivity to minimize false negatives (i.e., avoiding missed dementia cases). Therefore, we selected the more sensitive clinical cut‐off of ≥ 11. The consequence of an increased false‐positive rate at this threshold is clinically acceptable, as positive screens will reflexively trigger comprehensive diagnostic evaluations. However, these diagnostic accuracy estimates may be optimistic because they are derived from a selected, enriched clinical sample with clear‐cut diagnostic boundaries.

Furthermore, the ROC analysis demonstrated that the 6‐CIT also holds significant potential for identifying MCI (AUC = 0.976), with an optimal cutoff of ≥ 2 yielding both sensitivity and specificity of 96.7%. Multivariable logistic regression confirmed that this strong discrimination remained robust across different educational strata. While Abdel‐Aziz and Larner reported more modest accuracy for MCI (sensitivity 65.6%, specificity 70.3%; Abdel‐Aziz and Larner 2015), our higher accuracy is likely influenced by our strict exclusion criteria (e.g., systematically removing patients with GDS ≥ 8 and major comorbidities). This approach may have created an artificially clear distinction between the Normal and MCI groups, contributing to the high discrimination observed in our dataset.

The interpretation of these findings must consider several critical limitations. First, the use of convenience sampling and deliberately balanced diagnostic groups (*n* = 60 per group) does not reflect real‐world prevalence, potentially inflating diagnostic accuracy estimates, and the high diagnostic accuracy observed in this selected clinical sample must be generalized to routine primary care populations with caution. Second, the systematic exclusion of patients with depressive symptoms (GDS ≥ 8) introduced spectrum restriction, limiting the generalizability of the findings since depression frequently co‐occurs with cognitive complaints in older adults. Third, the test–retest subsample included only two cognitively normal participants, limiting the generalizability of stability estimates for healthy individuals. Fourth, although we statistically adjusted for the effect of education in regression models, specific normative corrections for educational background were not developed for the clinical cut‐off scores. Finally, the selected optimal cut‐offs lack external validation in an independent sample. Despite these caveats, the evidence suggests that the Persian 6‐CIT is a reliable cognitive screen. Its brevity (< 5 min) and ease of administration make it a promising tool. However, its practical utility and generalizability to broader settings, such as routine primary care or emergency departments, remain an extrapolation and should be formally evaluated in future unselected cohort studies.

## 5 Conclusion

The Persian 6‐CIT demonstrates favorable psychometric properties and high diagnostic discrimination in a specialized clinical sample of older adults. While it shows promise as an efficient cognitive screening instrument, external validation of the proposed cut‐offs in broader, unselected community and primary care populations is required before it can be firmly recommended to improve early case‐finding in routine clinical practice.

## Author Contributions

**Seyed Mohammad Mahdi Sadeghi**: investigation, formal analysis. **Amirreza Esmaeili**: formal analysis, writing – original draft, writing – review and editing. **Maryam Pourshams**: investigation. **Vahid Saeedi**: investigation, formal analysis. **Seyed Kazem Malakouti**: writing – review and editing, supervision. **Leila Kamalzadeh**: supervision, writing – original draft, writing – review and editing.

## Funding

The authors have nothing to report.

## Ethics Statement

The study protocol, including all procedures involving human participants, was reviewed and approved by the Ethics Committee of Iran University of Medical Sciences under the ethics code IR.IUMS.REC.1401.452. The research was conducted in strict accordance with the ethical principles outlined in the Declaration of Helsinki (World Medical Association 2013). Prior to participation, all individuals provided written informed consent. For participants categorized as having Major Neurocognitive Disorder, informed consent was obtained from their legally authorized representatives in cases where the participant lacked the capacity to consent. Confidentiality and anonymity of all participant data were maintained throughout the study.

## Conflicts of Interest

The authors have no conflict of interest to declare.

## Data Availability

The data that support the findings of this study are available on request from the corresponding author. The data are not publicly available due to privacy and ethical restrictions regarding patient confidentiality. However, to ensure full analytical traceability, the complete analytical code (syntax) detailing data processing, ROC analyses, and regression models is publicly available in a persistent open repository: https://osf.io/8vmgt.
